# Association Between Preoperative Gait Speed and Mortality in Patients with Transcatheter Edge-to-Edge Mitral Repair

**DOI:** 10.3390/diseases14070261

**Published:** 2026-07-20

**Authors:** Hirotaka Fukuda, Akihisa Sugawa, Takashi Miyamoto, Akira Nonoue, Terumi Fujimoto, Kazuki Tobita, Tomoyuki Arai

**Affiliations:** 1 Department of Rehabilitation, Saitama Prefectural Cardiovascular and Respiratory Center, Saitama 360-0197, Japan; sugawa.akihisa@saitama-pho.jp (A.S.); fujimoto.terumi@saitama-pho.jp (T.F.); 2 Department of Physical Therapy, Faculty of Health and Medical Care, Saitama Medical University, Saitama 350-8550, Japan; k_tobita@saitama-med.ac.jp; 3 Department of Cardiovascular Medicine, Saitama Prefectural Cardiovascular and Respiratory Center, Saitama 360-0197, Japan; miyamoto.takashi@saitama-pho.jp (T.M.); nonoue.akira@saitama-pho.jp (A.N.)

**Keywords:** gait speed, transcatheter edge-to-edge repair, mitral regurgitation, frailty, prognosis

## Abstract

Background: Transcatheter Edge-to-Edge Repair (TEER) is a therapeutic option established for older patients with heart failure and concomitant mitral regurgitation. Frailty is associated with prognosis after transcatheter valve interventions. However, despite clinical potential, evidence for gait speed as a simplified prognostic marker in patients undergoing TEER remains insufficient. Objectives: This study aimed to investigate the association between preoperative gait speed and mid- to long-term mortality after TEER. Methods: We conducted a single-center retrospective cohort study of 97 patients (mean age: 78.9 ± 8.7 years; 56.7% male) who survived the first 7 days after TEER and had available preoperative gait speed data. Preoperative gait speed was assessed, and clinical data were obtained from medical records. Cox regression analysis was performed to elucidate the association between preoperative gait speed and mortality. Results: In Cox proportional hazards models, higher gait speed (per 0.1 m/s increase) was associated with lower mortality after adjustment for the Society of Thoracic Surgeons risk score and handgrip strength (hazard ratio: 0.81; 95% confidence interval: 0.68–0.97; *p* = 0.02). In time-dependent receiver operating characteristic curve analysis, gait speed showed moderate discriminative ability for mortality, with area under the curve values of 0.749 at 1 year and 0.710 at 2 years. A gait speed of 0.8 m/s was used as an exploratory threshold for survival stratification, and patients with gait speed < 0.8 m/s had lower survival than those with gait speed ≥ 0.8 m/s (log-rank *p* < 0.01). Conclusions: Lower preoperative gait speed was associated with higher mid- to long-term mortality after TEER. Preoperative gait speed may provide clinically useful information for exploratory risk stratification, although the cutoff-based findings require external validation.

## 1. Introduction

Heart failure (HF) remains a major cause of mortality in Japan, ranking as the second leading cause of death [1]. The incidence of HF continues to rise, and this trend is expected to accelerate with population aging [2,3]. Mitral regurgitation (MR) is common in patients with HF and is associated with adverse clinical outcomes; in the BIOSTAT-CHF cohort, moderate-to-severe MR was observed in 41.1% of patients with worsening chronic or new-onset acute HF [4]. Although surgical intervention is an established treatment option for MR, the high operative risk associated with advanced age and comorbidities renders some patients ineligible for conventional surgery [5,6]. In this context, Transcatheter Edge-to-Edge Repair (TEER) has emerged as a minimally invasive therapeutic alternative for high-risk patients [7,8]. Clinical evidence regarding the impact of TEER on outcomes remains mixed; the COAPT trial demonstrated improvements in HF symptoms and prognosis [9], whereas the MITRA-FR trial reported no prognostic benefit [10]. As TEER has become more widely adopted, identifying predictors of clinical outcomes has become increasingly important, with frailty emerging as a key area of interest. Frailty is characterized by reduced physiological reserve and diminished resilience to stressors and encompasses physical, mental, and social dimensions of vulnerability [11,12]. Frailty is prevalent among older adults and has been associated with adverse postoperative outcomes [13,14]. Comprehensive geriatric assessment is commonly used to evaluate frailty, incorporating multidimensional factors into standardized scales [15,16]. Frailty is also frequently assessed using the Cardiovascular Health Study (CHS) phenotype, which includes slowness (gait speed) and weakness (handgrip strength) among its five components. Gait speed is a simple and reproducible measure and has been shown to predict a range of adverse outcomes [17,18]. Large-scale studies have demonstrated an association between slower gait speed and higher mortality in older adults [17]. Furthermore, gait speed has been identified as a useful marker for risk stratification in patients undergoing invasive cardiac procedures [17,19,20]. In transcatheter aortic valve implantation (TAVI), a percutaneous catheter-based procedure analogous to TEER, preoperative frailty and gait speed have been shown to be associated with postoperative outcomes [21,22]. Frailty has also been associated with worse outcomes in patients undergoing TEER [23]. However, previous studies have generally assessed frailty using multidomain measures, which may limit their routine implementation in preprocedural clinical practice. In contrast, gait speed is a simple, objective, and reproducible measure that can be assessed quickly without specialized equipment. Evidence focusing on preoperative gait speed as a single, readily implementable measure in contemporary TEER cohorts remains limited. Moreover, most previous evidence has been derived from Western cohorts and from earlier TEER eras. Whether a single physical performance measure such as gait speed has prognostic value in contemporary Asian TEER patients, who may differ in body size and sarcopenia profiles, remains unclear. Preoperative gait speed assessment may help risk stratification, inform shared decision-making, and guide peri-procedural management. This study aimed to investigate the association between preoperative gait speed and post-TEER survival in a single-center retrospective cohort and to evaluate the discriminative performance of gait speed for exploratory risk stratification.

## 2. Materials and Methods

### 2.1. Study Population

**Figure 1:**
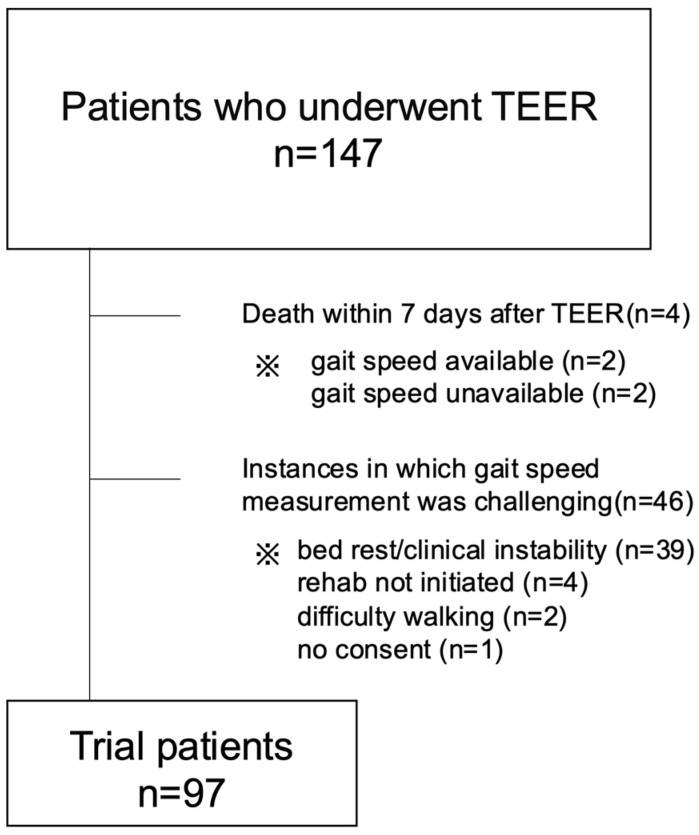
Flow diagram of the study. TEER, transcatheter edge-to-edge repair.

This was a single-center retrospective cohort study at the Saitama Prefectural Cardiovascular and Respiratory Center. Ethical approval was obtained from the Ethics Committee of the Saitama Prefectural Cardiovascular and Respiratory Center (approval number: 2024013), and the study was conducted in accordance with the Declaration of Helsinki. The requirement for written informed consent was waived by the Ethics Committee because of the retrospective nature of the study, and information regarding the study was disclosed to patients through an opt-out approach. We included consecutive patients who underwent TEER between July 2019 and March 2025. All TEER procedures were performed using the MitraClip™ (Abbott, Santa Clara, CA, USA) system. Echocardiographic parameters, including pre- and post-procedural LVEF, LAD, and LVEDD, were measured using standard transthoracic echocardiography systems manufactured by Philips (Amsterdam, The Netherlands), in accordance with guideline recommendations [24]. LVEF was assessed using the modified biplane Simpson’s method when feasible, and LAD and LVEDD were measured from standard parasternal long-axis views. MR severity was graded on a semi-quantitative scale from 1+ (mild) to 4+ (severe), based on an integrative echocardiographic assessment (color Doppler jet characteristics, vena contracta width, pulmonary vein flow patterns, and chamber remodeling), in accordance with guideline recommendations [25]. MR etiology was classified as degenerative MR, functional MR, or mixed MR based on echocardiographic findings and clinical records. Among patients diagnosed with FMR, FMR subtype was additionally classified as atrial FMR, ventricular FMR, or mixed FMR based on echocardiographic findings and clinical records. Atrial FMR was defined as FMR predominantly associated with left atrial enlargement and atrial fibrillation in the absence of marked left ventricular remodeling, whereas ventricular FMR was defined as FMR predominantly associated with left ventricular dilation and/or systolic dysfunction. Cases with overlapping atrial and ventricular features were classified as mixed FMR. For the primary analysis, we excluded patients who died within 7 days after TEER as early peri-procedural deaths, and patients in whom preoperative gait speed could not be assessed. The flow diagram of this study is shown in Figure 1.

### 2.2. Measurements

Clinical data were collected from medical records. Data on age, sex, body mass index (BMI), New York Heart Association (NYHA) functional class, MR etiology and severity, echocardiographic parameters (pre- and post-procedural left ventricular ejection fraction [LVEF], left atrial diameter [LAD], and left ventricular end-diastolic diameter [LVEDD]), and laboratory data were collected. TEER procedural variables, including procedure urgency, procedure time, and blood loss, were also extracted. Surgical risk was assessed using the Society of Thoracic Surgeons (STS) predicted risk of mortality score for mitral valve replacement [26]. The STS risk score was calculated by attending cardiologists at the time when TEER was planned, using the most recent preoperative clinical data available at that time. The calculation was based on the patients’ preoperative clinical status and medical records, including demographic characteristics, comorbidities, functional status, and cardiac condition. In this study, the STS risk score for mitral valve replacement was used as a comprehensive summary measure of baseline surgical risk rather than as a TEER-specific risk score. In-hospital variables included length of hospital stay, days to first standing, days to walking, days to 100 m walking, and maximum continuous walking distance were obtained from physical therapy records. Data on heart failure medications at discharge were additionally collected from medical records. Medications were categorized as renin–angiotensin system inhibitors, mineralocorticoid receptor antagonists, angiotensin receptor–neprilysin inhibitors, diuretics, sodium–glucose cotransporter 2 inhibitors, beta-blockers, and other heart failure medications. Data on heart failure rehospitalization within 1 year after TEER were also collected when available. Heart failure rehospitalization was defined as rehospitalization for worsening heart failure that could be confirmed through rehospitalization at our institution, outpatient medical records, interviews during follow-up visits, or telephone follow-up.

### 2.3. Physical and Cognitive Function

Preoperative physical and cognitive function was assessed using gait speed, handgrip strength, the Mini-Mental State Examination (MMSE), and the Clinical Frailty Scale (CFS). Gait speed and handgrip strength were assessed as objective physical performance measures and as components of the physical frailty phenotype [13]. MMSE was used as a global cognitive screening tool [27], and CFS was used to evaluate overall frailty based on the patient’s premorbid functional status [28]. These assessments were performed by physicians or physical therapists within 7 days before TEER. Gait speed was measured over a 5 m walkway with a 3 m acceleration and deceleration zone. The time to traverse the central 5 m segment (from 3 to 8 m) was recorded to calculate gait speed. Patients were allowed to use their usual walking aids when needed. Physical assistance was provided only when required for safety and not to increase walking speed. Oxygen supplementation, when clinically required, was continued during the assessment under the same condition as during usual preoperative mobilization. Two trials were performed, and the faster value was used for analysis to reduce the influence of occasional underperformance related to unfamiliarity with the measurement procedure in this older patient population. Histories of orthopedic disease and stroke were additionally collected from medical records as factors potentially affecting gait speed. Handgrip strength was measured in the sitting position using a Smedley dynamometer (T.K.K. 5401; Takei Scientific Instruments Co., Ltd., Niigata, Japan). Two trials were performed for each hand, and the maximum value across all trials was recorded. MMSE was administered using a standardized questionnaire. CFS was determined based on interviews regarding the patients’ premorbid living situation immediately prior to hospitalization.

### 2.4. Survival Status and Follow-Up

Post-TEER survival status was obtained from medical records, scheduled outpatient visits, and telephone follow-up. For surviving patients, the final follow-up date was defined as the last documented clinic visit or telephone contact. Mortality was verified through hospital records, telephone follow-up after missed appointments, or reports from family members.

### 2.5. Outcome

The primary outcome was mid- to long-term all-cause mortality after TEER, defined as death occurring from day 8 onward after the procedure. The primary analysis evaluated the association between preoperative gait speed and time to death among patients who survived the first 7 days after TEER. Early deaths within 7 days after TEER were considered clinically important peri-procedural outcomes; however, they were not included in the primary outcome because they may be more strongly influenced by procedure-related complications, acute hemodynamic changes, and peri-procedural management than by the chronic physical vulnerability reflected by preoperative gait speed. These early deaths were reported separately in the patient flow. As an exploratory analysis, we evaluated the discriminative performance of preoperative gait speed for mid- to long-term mortality and examined survival differences using an exploratory gait speed threshold.

### 2.6. Statistical Analysis

Categorical variables are presented as absolute values and percentages, and continuous variables as means ± standard deviations (SDs) or medians (interquartile ranges). Missing values were not imputed, and analyses were performed using available data for each variable. The number of available observations for variables with missing data is shown in the table footnotes. To identify factors associated with mortality, we performed univariable and multivariable Cox proportional hazards regression analyses. Gait speed was scaled per 0.1 m/s increase to enhance clinical interpretability. The proportional hazards assumption was assessed by testing time-dependent covariate effects. Interaction terms between each covariate included in the Cox models and the natural logarithm of survival time were entered into the models. Non-significant interaction terms were interpreted as supporting the proportional hazards assumption. Multivariable models were prespecified based on clinical relevance and to minimize overfitting given the limited number of events. Accordingly, we prioritized clinical interpretability and model parsimony over full adjustment. We fitted two multivariable models to examine the robustness of the association: Model 1 adjusted for the STS risk score, and Model 2 additionally adjusted for handgrip strength. Model 1 was adjusted for the STS risk score for mitral valve replacement because it is a comprehensive surgical risk metric incorporating key prognostic factors including age and sex. Model 2 additionally adjusted for handgrip strength to test the robustness of the association between gait speed and mortality after accounting for another core physical frailty component (weakness) included in the CHS phenotype. To further examine whether the association between gait speed and mortality was robust to adjustment for individual clinical factors, we constructed an exploratory alternative model (Model 3) without the STS risk score. Covariates for this model were selected based on the results of univariable analyses and their anticipated clinical relevance to mortality after TEER. Model 3 included age, hemoglobin, eGFR, procedure urgency, and the Clinical Frailty Scale. Because of the limited number of events, this model was intended as an exploratory analysis rather than a fully adjusted model. Other potential confounders, including MR etiology and additional echocardiographic or procedural variables, were not included simultaneously to avoid model overfitting and excessive overlap among covariates. To evaluate the incremental prognostic value of gait speed, we compared nested Cox models with and without gait speed using likelihood-ratio tests. Model fit was assessed by the change in −2 log likelihood after adding gait speed to each base model. To evaluate the discriminative performance of gait speed for all-cause mortality while accounting for variable follow-up duration and censoring, time-dependent receiver operating characteristic (ROC) curve analysis was performed at clinically meaningful time points of 1 and 2 years after TEER. The time-dependent area under the curve (AUC) was estimated using inverse probability of censoring weighting. Because lower gait speed indicates a higher risk of mortality, gait speed was analyzed in the reversed direction for ROC estimation. Exploratory cutoff values were estimated based on the balance between sensitivity and specificity at each time point. Considering both the time-dependent ROC results and previously reported gait speed thresholds in older adults, a gait speed of 0.8 m/s was used as an exploratory threshold for survival stratification. This threshold was not interpreted as a clinically established TEER-specific cutoff. Patients were then stratified based on this exploratory threshold, with cumulative survival compared across groups using Kaplan–Meier curves and the log-rank test. Baseline characteristics were compared between patients included in the final analysis and those excluded to assess potential selection bias. To further assess potential selection bias related to gait speed availability, patients included in the final analysis were compared with patients in whom preoperative gait speed could not be assessed among those who survived beyond 7 days after TEER. Cumulative survival was compared between these groups using Kaplan–Meier curves and the log-rank test. Most statistical analyses were performed using SPSS version 31.0.1.0, and time-dependent ROC analysis was performed using R version 4.6.1 with the timeROC package (version 0.4.1). A two-sided *p* value < 0.05 was considered statistically significant.

## 3. Results

During the study period, 147 patients underwent TEER. Four patients who died within 7 days after TEER were excluded from the primary analysis as early peri-procedural deaths; preoperative gait speed was available in two of these patients and unavailable in the remaining two. Among the 143 patients who survived beyond 7 days after TEER, 46 patients were excluded because preoperative gait speed could not be assessed. Consequently, 97 patients were included in the final analysis (Figure 1). To assess potential selection bias related to gait speed availability, baseline characteristics and subsequent mortality were compared between the final analysis cohort and the gait speed-unavailable subgroup among patients who survived beyond 7 days after TEER (Table S1). Compared with the final analysis cohort, the gait speed-unavailable subgroup was younger and had a higher proportion of NYHA class IV and urgent procedures, higher BNP and STS score for MVR, lower hemoglobin and albumin levels, and higher eGFR. No statistically significant difference was observed in preoperative LVEF between the groups. Subsequent mortality occurred in 11 of 46 patients (23.9%) in the gait speed-unavailable subgroup and in 31 of 97 patients (32.0%) in the final analysis cohort. Kaplan–Meier analysis showed no significant difference in survival between the two groups (log-rank *p* = 0.92; Supplementary Figure S2). Baseline characteristics and clinical outcomes of the final analysis cohort are summarized in Table 1.

The mean age was 78.9 ± 8.7 years, and 55 patients (56.7%) were male. Histories of orthopedic disease and stroke were observed in 20 patients (20.6%) and 5 patients (5.2%), respectively. A history of orthopedic disease was more frequent in the lower gait speed group than in the higher gait speed group (31.6% vs. 13.6%, *p* = 0.03), whereas a history of stroke did not differ significantly between groups. The majority of patients had functional MR (76.3%) and severe MR (grade 3+–4+) at baseline (97.9%), with a mean LVEF of 39.0 ± 15.4%. Among patients diagnosed with FMR, atrial FMR, ventricular FMR, and mixed FMR were observed in 8 (10.8%), 54 (73.0%), and 12 (16.2%) patients, respectively. The distribution of FMR subtype did not differ significantly between the lower and higher gait speed groups (*p* = 0.94). The median follow-up duration was 713.0 days (interquartile range: 322.0–1088.5 days). Heart failure medications at discharge are shown in Table 1. The use of renin–angiotensin system inhibitors, mineralocorticoid receptor antagonists, angiotensin receptor–neprilysin inhibitors, diuretics, sodium–glucose cotransporter 2 inhibitors, beta-blockers, and other heart failure medications did not differ significantly between the lower and higher gait speed groups. Heart failure rehospitalization within 1 year after TEER, confirmed through available follow-up information, occurred in 17 patients (17.5%). The proportion of heart failure rehospitalization was numerically higher in the lower gait speed group than in the higher gait speed group (25.6% vs. 11.1%), although the difference did not reach statistical significance (*p* = 0.06). During follow-up, 31 deaths occurred. The causes of death were cardiovascular death in 14 patients (45.2%), non-cardiovascular death in 8 patients (25.8%), and unknown or undetermined death in 9 patients (29.0%); heart failure was the most common cardiovascular cause of death (11 of 14 cardiovascular deaths), and malignancy was the most common non-cardiovascular cause of death (5 of 8 non-cardiovascular deaths) (Table S2). The Kaplan–Meier estimated survival was 87.8% at 1 year and 73.5% at 2 years (Figure S1).

### 3.1. Factors Associated with Survival Time

Associations between candidate predictors and mortality are shown in Table 2. In the univariable analysis, higher preoperative gait speed (per 0.1 m/s increase) was associated with lower risk of mortality (hazard ratio [HR]: 0.72; 95% confidence interval [CI]: 0.62–0.84; *p* < 0.01). Among the other physical and cognitive measures, handgrip strength (HR: 0.92; 95% CI: 0.88–0.97; *p* < 0.01) and CFS (HR: 1.55; 95% CI: 1.21–2.00; *p* < 0.01) were also associated with mortality in the univariable analysis, whereas MMSE was not significantly associated with mortality (HR: 0.93; 95% CI: 0.85–1.01; *p* = 0.09). In the multivariable models, gait speed remained associated with mortality after adjustment for the STS risk score (Model 1; HR: 0.77; 95% CI: 0.66–0.90; *p* < 0.01) and after additional adjustment for handgrip strength (Model 2; HR: 0.81; 95% CI: 0.68–0.97; *p* = 0.02). In the exploratory alternative model without the STS risk score, gait speed remained significantly associated with mortality after adjustment for age, hemoglobin, eGFR, procedure urgency, and CFS (Model 3; HR: 0.69; 95% CI: 0.54–0.88; *p* < 0.01). As a sensitivity analysis, we repeated the Cox regression analysis including the two patients who died within 7 days after TEER but had available preoperative gait speed data. The association between gait speed and mortality was materially unchanged (Table S3). No statistically significant time-dependent effects were observed for gait speed or the covariates included in the multivariable models (all *p* > 0.05), supporting the proportional hazards assumption. In likelihood-ratio tests comparing nested Cox models, adding gait speed significantly improved model fit in Model 1 (Δχ^2^ = 13.18, df = 1, *p* < 0.001), Model 2 (Δχ^2^ = 7.27, df = 1, *p* = 0.007), and Model 3 (Δχ^2^ = 9.54, df = 1, *p* = 0.002).

### 3.2. Determination of Cutoff Values

**Figure 2:**
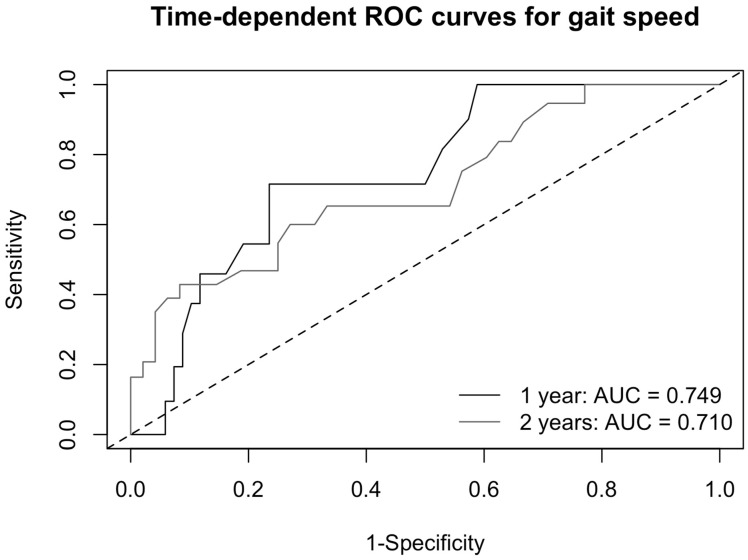
Time-dependent receiver operating characteristic curves for gait speed predicting all-cause mortality at 1 and 2 years after transcatheter edge-to-edge repair. The time-dependent AUC values were 0.749 at 1 year and 0.710 at 2 years. AUC, area under the curve; ROC, receiver operating characteristic.

The discriminative performance of gait speed for all-cause mortality was evaluated using time-dependent ROC analysis (Figure 2). The time-dependent AUCs for gait speed were 0.749 at 1 year and 0.710 at 2 years after TEER, indicating moderate discriminative ability. The corresponding exploratory cutoff values were 0.77 m/s at 1 year, with a sensitivity of 0.727 and a specificity of 0.768, and 0.79 m/s at 2 years, with a sensitivity of 0.619 and a specificity of 0.729. Based on these results and previously reported gait speed thresholds in older adults, 0.8 m/s was used as an exploratory threshold for subsequent descriptive survival stratification.

### 3.3. Survival Stratification

**Figure 3:**
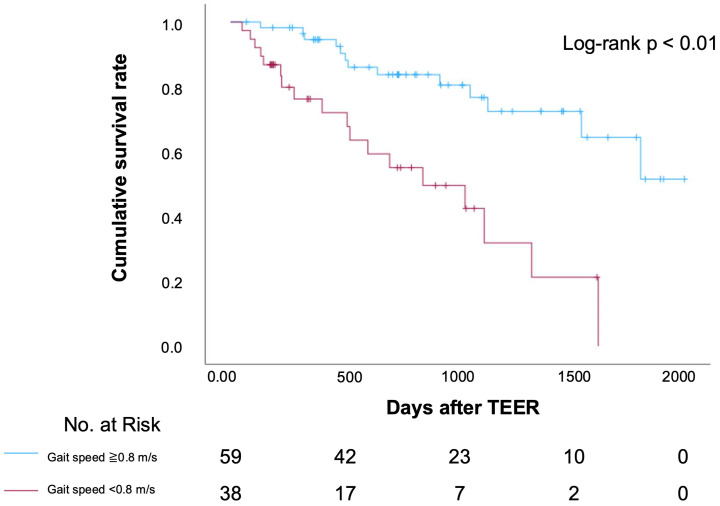
Kaplan–Meier curves for all-cause mortality according to gait speed group stratified by the exploratory threshold of 0.8 m/s. Censoring marks are shown on the curves. The median follow-up duration was 772.0 days (interquartile range: 465.0–1366.0 days) in the gait speed ≥ 0.8 m/s group and 347.0 days (interquartile range: 183.0–860.0 days) in the gait speed < 0.8 m/s group. TEER, transcatheter edge-to-edge repair.

Patients were stratified into two groups according to the exploratory threshold of 0.8 m/s. In Kaplan–Meier curves, patients with lower gait speed (<0.8 m/s) had worse survival compared with those with higher gait speed (≥0.8 m/s) (Figure 3; log-rank *p* < 0.01).

## 4. Discussion

This single-center retrospective cohort study showed that lower preoperative gait speed was associated with an increased risk of mortality after TEER among patients who survived the early peri-procedural period. Furthermore, exploratory survival stratification using a gait speed threshold of 0.8 m/s showed worse survival in patients with lower gait speed. These findings suggest that preoperative gait speed as a simple physical performance measure may provide clinically useful information for risk assessment among TEER candidates in an exploratory context.

### 4.1. Post-TEER Survival

Large-scale studies, including the COAPT [9] and EVEREST II [8] trials, have reported post-TEER outcomes, and the participants of these reports were patients around 70 years of age. Compared with these trials, our cohort included a more clinically high-risk population, with patients who were older (mean age: 78.9 years) and had lower [8] or comparable [9] cardiac function (mean LVEF: 39.0%). Despite these characteristics, the overall survival in our cohort was comparable to that reported previously [8,9]. However, differences in patient selection, MR etiology, baseline HF severity, and procedural indications should be considered when interpreting these comparisons. Because most patients in this cohort had FMR, we additionally examined FMR subtype; although atrial FMR is recognized as a distinct clinical entity related to left atrial remodeling and atrial fibrillation [29], FMR subtype did not differ significantly between gait speed groups, and the small number of atrial FMR cases limited further evaluation of its prognostic impact.

### 4.2. Association Between Gait Speed and Mortality Risk

Gait speed is a well-established marker of physical performance and overall health status in older adults [17]. In the present study, lower gait speed was associated with a higher risk of post-TEER mortality, and this association persisted after adjustment for the STS risk score and handgrip strength, as well as in an exploratory alternative model including selected clinical covariates such as hemoglobin, renal function, procedure urgency, and CFS. Because handgrip strength is another core component of the physical frailty phenotype, the persistence of the association after adjustment for handgrip strength suggests that gait speed may reflect prognostically relevant vulnerability not fully captured by muscle strength alone. Among the other physical and cognitive measures, handgrip strength and CFS were associated with mortality in the univariable analysis, whereas MMSE was not. The association between CFS and mortality is consistent with previous evidence indicating the prognostic importance of frailty in patients undergoing TEER [23]. Taken together, these findings do not diminish the value of multidomain frailty assessment, but rather suggest that gait speed may provide additional and clinically practical information as a single physical performance measure. Thus, the present study extends previous work on frailty in TEER patients by showing that preoperative gait speed may capture prognostically relevant vulnerability even when considered alongside other frailty-related measures.

Gait speed may integrate multiple dimensions of impaired health status, including cardiac functional limitation, skeletal muscle dysfunction, nutritional status, comorbidity burden, and overall physiological reserve. This interpretation is consistent with previous studies suggesting that preoperative physical performance reflects physiological reserve and vulnerability to peri-procedural stress, which may influence longer-term outcomes after valvular interventions. Therefore, gait speed should be interpreted as a practical component of comprehensive risk assessment rather than as a substitute for multidomain frailty assessment.

In the present study, time-dependent ROC analysis demonstrated moderate discriminative ability of gait speed for mortality at 1 and 2 years after TEER. The exploratory cutoff values derived from the time-dependent ROC analysis were close to 0.8 m/s, a commonly used gait speed threshold in older adults [17] and in patients undergoing transcatheter cardiac interventions [21]. Therefore, we used 0.8 m/s as an exploratory threshold for descriptive survival stratification. However, the cutoff-based analysis should be regarded as supportive and exploratory, whereas the primary finding of this study is the association between gait speed as a continuous variable and mortality. The 0.8 m/s threshold should not be interpreted as a clinically established TEER-specific cutoff, and external validation is required before clinical application.

### 4.3. Peri-Procedural Management and Rehabilitation

Our results do not demonstrate a causal relationship between improving gait speed and improved post-TEER survival. Rather, preoperative gait speed should be interpreted as a practical marker of overall vulnerability. In patients with advanced HF or those requiring urgent procedures, the feasibility of structured preoperative rehabilitation may be limited. Therefore, gait speed may help identify patients who require tailored peri-procedural strategies, including multidisciplinary rehabilitation, early mobilization, and structured transition-to-care planning. Prospective studies are needed to determine whether interventions targeting physical performance or changes in gait speed after TEER can improve clinical outcomes.

### 4.4. Clinical Implications

Preoperative gait speed assessment is simple, rapid, and readily implementable in routine practice, and our findings suggest that gait speed may contribute to risk stratification in patients considered for TEER. Notably, even within this relatively stable subgroup, gait speed remained independently associated with mortality. Incorporating gait speed into a comprehensive clinical assessment may help clinicians identify vulnerable patients who may require intensive follow-up and additional supportive care after TEER. We additionally examined heart failure medications at discharge and heart failure rehospitalization within 1 year after TEER. The use of major heart failure medication classes did not differ significantly between gait speed groups, suggesting that differences in discharge medication were unlikely to fully explain the observed association between gait speed and mortality. Heart failure rehospitalization was numerically more frequent in the lower gait speed group, although the difference was not statistically significant. This finding may suggest that lower gait speed reflects a broader vulnerability to adverse clinical outcomes after TEER; however, this result should be interpreted cautiously because rehospitalization events were collected only when they could be confirmed through available follow-up information.

### 4.5. Limitations

This study has several limitations. First, as a retrospective single-center study, the findings may have limited generalizability. Second, because of the limited number of events, we prespecified parsimonious multivariable models to mitigate overfitting; accordingly, the results should be considered exploratory. Although we additionally constructed an exploratory alternative model including selected clinical covariates, the number of events limited the extent of multivariable adjustment. Therefore, residual confounding from factors such as nutritional status, renal function, heart failure severity, procedure urgency, MR etiology, and frailty cannot be fully excluded. Third, gait speed could not be assessed in a substantial proportion of patients, most commonly due to pre-procedural clinical instability. Although survival did not differ significantly between the final analysis cohort and the gait speed-unavailable subgroup among patients surviving beyond 7 days after TEER, the inability to assess gait speed may still reflect clinical instability and introduce potential selection bias. Therefore, our findings should be interpreted as primarily applicable to ambulatory and clinically stable TEER candidates who are able to undergo preoperative gait speed assessment. In addition, patients who died within 7 days after TEER were excluded from the primary analysis because the primary outcome was defined as mid- to long-term mortality after TEER. Although this definition was chosen to focus on mortality beyond the early peri-procedural period, early peri-procedural deaths are clinically important outcomes, and survivor bias cannot be fully excluded. Therefore, the present findings should not be interpreted as describing the association between gait speed and total mortality immediately after TEER. Fourth, although the exploratory threshold of 0.8 m/s was supported by the time-dependent ROC-derived cutoff values, the cutoff-based analysis was derived from a single-center cohort with a limited number of events. Therefore, this threshold requires external validation and should not be applied as a definitive TEER-specific clinical cutoff. Fifth, information on heart failure rehospitalization within 1 year after TEER was limited to events confirmed through rehospitalization at our institution, outpatient records, interviews, or telephone follow-up; therefore, rehospitalization events, particularly those occurring at other institutions, may have been underestimated. Finally, changes in gait speed after TEER were not systematically assessed, and future studies should evaluate whether serial changes in gait speed provide additional prognostic information beyond preoperative gait speed.

## 5. Conclusions

In this single-center retrospective cohort study, lower preoperative gait speed was associated with an increased risk of mid- to long-term mortality after TEER. Preoperative gait speed may serve as a practical measure to support exploratory risk stratification and peri-procedural planning. Prospective multicenter studies are warranted to confirm these findings and to clarify whether physical performance assessment can inform post-TEER management and improve risk stratification.

## Figures and Tables

**Table 1:** Patient background characteristics.

Factor	Overall	Gait Speed ≥ 0.8 m/s	Gait Speed < 0.8 m/s	*p* Value
	(*n* = 97)	(*n* = 59)	(*n* = 38)	
Characteristics				
Age (years)	78.9 ± 8.7	77.2 ± 8.5	81.4 ± 8.3	0.02 *
Sex [male (%)]	55 (56.7)	36 (61.0)	19 (50.0)	0.29
BMI (kg/m^2^)	20.8 ± 3.1	20.8 ± 3.1	20.7 ± 3.1	0.86
Hb (g/dL)	12.3 ± 1.9	12.4 ± 2.1	12.1 ± 2.1	0.52
BNP (pg/mL)	318.0 (180.3–589.6)	319.5 (201.1–547.5)	251.1 (134.7–898.2)	0.96
Cr (mg/dL)	1.68 ± 1.24	1.74 ± 1.36	1.59 ± 1.04	0.56
eGFR (mL/min/1.73 m^2^)	36.7 ± 16.4	37.0 ± 16.4	36.3 ± 16.6	0.84
Alb (g/dL)	3.65 ± 0.44	3.75 ± 0.39	3.49 ± 0.48	<0.01 **
History of orthopedic disease [n (%)]	20 (20.6)	8 (13.6)	12 (31.6)	0.03 *
History of stroke [*n* (%)]	5 (5.2)	2 (3.4)	3 (7.9)	0.33
Preoperative Physical and Cognitive Function				
Gait Speed (m/s)	0.84 ± 0.22	0.97 ± 0.13	0.63 ± 0.16	<0.01 **
Grip Strength (kg)	23.1 ± 8.9	25.5 ± 8.5	19.4 ± 8.2	<0.01 **
MMSE (points)	25.0 ± 4.0	25.5 ± 3.4	24.2 ± 4.6	0.11
CFS [*n* (%)]				0.01 *
1	1 (1.0)	1 (1.7)	0 (0.0)	
2	6 (6.2)	6 (10.2)	0 (0.0)	
3	46 (47.4)	32 (54.2)	14 (37.8)	
4	24 (24.7)	14 (23.7)	10 (27.0)	
5	7 (7.2)	4 (6.8)	3 (8.1)	
6	10 (10.3)	2 (3.4)	8 (21.6)	
7	2 (2.1)	0 (0.0)	2 (5.4)	
Preoperative Clinical Background				
NYHA [*n* (%)]				0.27
I	5 (5.2)	5 (8.5)	0 (0.0)	
II	42 (43.3)	25 (42.4)	17 (44.7)	
III	28 (28.9)	15 (25.4)	13 (34.2)	
IV	22 (22.7)	14 (23.7)	8 (21.1)	
Type of MR [*n* (%)]				0.71
FMR	74 (76.3)	46 (78.0)	28 (73.8)	
DMR	11 (11.3)	7 (11.9)	4 (10.5)	
Mixed	12 (12.4)	6 (10.2)	6 (15.8)	
FMR subtype among patients with FMR [*n* (%)]				0.94
Atrial FMR	8 (10.8)	5 (10.9)	3 (10.7)	
Ventricular FMR	54 (73.0)	33 (71.7)	21 (75.0)	
Mixed/indeterminate FMR	12 (16.2)	8 (17.4)	4 (14.3)	
Severity of Preoperative MR [*n* (%)]				0.53
1+	0 (0.0)	0 (0.0)	0 (0.0)	
2+	2 (2.1)	2 (3.4)	0 (0.0)	
3+	25 (26.0)	15 (25.4)	10 (27.0)	
4+	69 (71.9)	42 (71.2)	27 (73.0)	
STS score for MVR (%)	9.88 ± 6.18	8.60 ± 4.69	11.79 ± 7.58	0.01 *
Preoperative LVEF (%)	39.0 ± 15.4	38.6 ± 14.8	39.7 ± 16.5	0.74
Preoperative LAD (mm)	50.7 ± 8.1	50.2 ± 8.1	51.5 ± 8.1	0.45
Preoperative LVEDD (mm)	61.1 ± 12.5	60.7 ± 10.4	61.6 ± 15.4	0.73
Operative Details and Postoperative Cardiac Function				
Operation Time (min)	81.0 (59.5–101.5)	78.0 (59.0–100.0)	79.5 (55.8–104.0)	0.99
Urgency [Planned (%)]	51 (53.7)	36 (63.2)	15 (39.5)	0.02 *
Blood Loss (mL)	20.0 (5.0–50.0)	20.0 (5.0–50.0)	20.0 (10.0–50.0)	0.68
Postoperative LVEF (%)	37.7 ± 15.2	37.1 ± 14.7	38.7 ± 16.1	0.62
Postoperative LAD (mm)	49.2 ± 8.6	48.6 ± 8.6	50.0 ± 8.7	0.45
Postoperative LVEDD (mm)	58.1 ± 11.1	58.8 ± 11.6	57.0 ± 10.4	0.43
Medications at discharge				
RAS inhibitors [*n* (%)]	39 (40.2)	25 (42.4)	14 (36.8)	0.59
MRA [*n* (%)]	68 (70.1)	42 (71.2)	26 (68.4)	0.77
ARNI [*n* (%)]	41 (42.3)	26 (44.1)	15 (39.5)	0.66
Diuretics [*n* (%)]	75 (77.3)	44 (74.6)	31 (81.6)	0.42
SGLT2 inhibitors [*n* (%)]	49 (50.5)	34 (57.6)	15 (39.5)	0.08
Beta-blockers [*n* (%)]	84 (86.6)	49 (83.1)	35 (92.1)	0.20
Other heart failure medications [*n* (%)]	53 (54.6)	34 (57.6)	19 (50.0)	0.46
Postoperative Hospitalization Details				
Hospital Stay (days)	9.0 (7.0–14.0)	9.0 (7.0–11.0)	12.5 (7.0–17.3)	0.02 *
Outcome [Discharge to Home (%)]	87 (89.7)	58 (98.3)	29 (76.3)	<0.01 **
Days to Standing (days)	1.0 (1.0–1.8)	1.0 (1.0–1.0)	1.0 (1.0–2.0)	0.28
Days to Walking (days)	1.0 (1.0–2.0)	1.0 (1.0–2.0)	1.0 (1.0–2.0)	0.26
Days to 100 m Walking (days)	2.0 (1.0–5.0)	2.0 (1.0–5.0)	2.0 (1.0–6.3)	0.20
Continuous Walking Distance (m)	100.0 (100.0–300.0)	200.0 (100.0–300.0)	100.0 (100.0–212.5)	<0.01 **

Continuous variables were analyzed using the *t*-test or Mann–Whitney U test, as appropriate, and categorical variables were compared using the chi-square test or Fisher’s exact test, as appropriate. Data are presented as mean ± standard deviation, median (interquartile range), or *n* (%), as applicable. * *p* < 0.05; ** *p* < 0.01. Alb, albumin; BMI, body mass index; BNP, brain natriuretic peptide; CFS, Clinical Frailty Scale; Cr, creatinine; DMR, degenerative mitral regurgitation; eGFR, estimated glomerular filtration rate; FMR, functional mitral regurgitation; Hb, hemoglobin; LAD, left atrial diameter; LVEDD, left ventricular end-diastolic diameter; LVEF, left ventricular ejection fraction; MMSE, Mini-Mental State Examination; MR, mitral regurgitation; MVR, mitral valve replacement; NYHA, New York Heart Association; STS, Society of Thoracic Surgeons. ARNI, angiotensin receptor–neprilysin inhibitor; MRA, mineralocorticoid receptor antagonist; RAS, renin–angiotensin system; SGLT2, sodium–glucose cotransporter 2. The number of available observations was 97 for all variables unless otherwise indicated. Available observations were as follows: albumin, *n* = 96; preoperative MR severity, *n* = 92; STS score for MVR, *n* = 95; Procedure urgency, *n* = 95; postoperative LAD, *n* = 96; days to standing, *n* = 96; days to walking, *n* = 96; days to 100 m walking, *n* = 84; and continuous walking distance, *n* = 91. Missing values were not imputed.

**Table 2:** Cox proportional hazard regression analysis for the primary outcome.

Factor	Univariable Analysis		Multivariable Analysis (Model 1)		Multivariable Analysis (Model 2)		Multivariable Analysis (Model 3)	
	Hazard Ratio	*p* Value	Hazard Ratio	*p* Value	Hazard Ratio	*p* Value	Hazard Ratio	*p* Value
Baseline Characteristics								
Age	1.06 (1.01–1.11)	0.02 *					1.04 (0.98–1.09)	0.19
Sex	1.91 (0.95–3.84)	0.07						
BMI	0.91 (0.81–1.02)	0.11						
BMI Category								
Low Weight (<18.5)	2.02 (0.97–4.21)	0.06						
Obesity (≥25)	0.93 (0.22–4.05)	0.93						
Hb	0.69 (0.56–0.86)	<0.01 **					0.80 (0.61–1.05)	0.11
BNP	1.00 (1.00–1.00)	0.36						
Cr	1.18 (0.96–1.46)	0.12						
eGFR	0.98 (0.95–1.00)	0.03 *					0.97 (0.94–1.00)	0.05
Alb	0.50 (0.25–1.00)	0.05 *						
Preoperative Physical and Cognitive Function								
Gait Speed (per 0.1 m/s)	0.72 (0.62–0.84)	<0.01 **	0.77 (0.66–0.90)	<0.01 **	0.81 (0.68–0.97)	0.02 *	0.69 (0.54–0.88)	<0.01 **
Grip Strength	0.92 (0.88–0.97)	<0.01 **			0.97 (0.92–1.01)	0.16		
MMSE	0.93 (0.85–1.01)	0.09						
CFS	1.55 (1.21–2.00)	<0.01 **					0.90 (0.62–1.30)	0.56
Preoperative Clinical Background								
NYHA	1.13 (0.76–1.67)	0.55						
Type of MR		0.51						
FMR	1.00 (reference)	-						
DMR	0.47 (0.11–2.00)	0.31						
Mixed	1.10 (0.32–3.70)	0.88						
STS score for MVR	1.11 (1.07–1.16)	<0.01 **	1.10 (1.04–1.15)	<0.01 **	1.10 (1.04–1.15)	<0.01 **		
Severity of Preoperative MR	1.83 (0.85–3.49)	0.13						
Preoperative LVEF	1.01 (0.98–1.03)	0.64						
Preoperative LAD	1.04 (0.99–1.09)	0.09						
Preoperative LVEDD	1.01 (0.98–1.04)	0.40						
Operative Details and Postoperative Cardiac Function								
Operation Time	1.01 (1.01–1.02)	0.01 *						
Urgency	1.44 (0.72–2.86)	0.30					0.97 (0.44–2.12)	0.93
Blood Loss	1.01 (1.01–1.02)	<0.01 **						
Postoperative LVEF	1.01 (0.98–1.03)	0.64						
Postoperative LAD	1.04 (1.00–1.08)	0.08						
Postoperative LVEDD	1.00 (0.97–1.03)	0.84						
Postoperative Hospitalization Details								
Hospital Stay	1.04 (1.02–1.05)	<0.01 **						
Outcome	8.22 (3.64–18.59)	<0.01 **						
Days to Standing	1.12 (1.01–1.23)	0.03 *						
Days to Walking	1.06 (1.02–1.09)	<0.01 **						
Days to 100 m Walking	1.06 (1.00–1.12)	0.05						
Continuous Walking Distance	1.00 (0.99–1.00)	0.01 *						

Hazard ratios are presented with 95% confidence intervals. * *p* < 0.05; ** *p* < 0.01. Model 1 was adjusted for the STS score for MVR; Model 2, for the STS score for MVR and handgrip strength; and Model 3, for age, hemoglobin, eGFR, procedure urgency, and CFS. Gait speed was analyzed per 0.1 m/s increase. The proportional hazards assumption was assessed using time-dependent covariate effects, and no significant time-dependent effects were observed. Likelihood-ratio tests showed that adding gait speed improved model fit in Model 1 (Δχ^2^ = 13.18, df = 1, *p* < 0.001), Model 2 (Δχ^2^ = 7.27, df = 1, *p* = 0.007), and Model 3 (Δχ^2^ = 9.54, df = 1, *p* = 0.002). Univariable Cox regression analyses were performed using available data for each variable. Missing values were not imputed.

## Data Availability

The data supporting the findings of this study are available from the corresponding author upon reasonable request.

## Supplementary Materials

The following supporting information can be downloaded at: https://www.mdpi.com/article/10.3390/diseases14070261/s1, Figure S1: Kaplan–Meier curve for all-cause mortality; Figure S2: Kaplan–Meier curves for all-cause mortality according to availability of preoperative gait speed assessment among patients surviving beyond 7 days after TEER; Table S1: Comparison between the final analysis cohort and the gait speed-unavailable subgroup among patients surviving beyond 7 days after TEER; Table S2: Causes of Death During Follow-up; Table S3: Sensitivity analysis including patients who died within 7 days after TEER and had available preoperative gait speed data.
