# Supplementary material for: Association Between Preoperative Gait Speed and Mortality in Patients with Transcatheter Edge-to-Edge Mitral Repair
Source: Diseases. 2026 Jul 20;14(7):261. doi: 10.3390/diseases14070261 (PMC13408858; doi:10.3390/diseases14070261)
Supplement: Supplementary file 1 [file diseases-14-00261-s001.zip › diseases-4393154-supplementary.pdf]

Table S1. Comparison between the final analysis cohort and the gait speed-unavailable subgroup among patients surviving beyond 7 days after TEER.

Factor	Overall (n=143)	Study patients (n=97)	Gait speed-unavailable subgroup (n=46)	P value
Characteristics				
Age (years)	77.7 ± 9.3	78.9 ± 8.7	75.2 ± 10.3	0.04*
Sex [male (%)]	84 (58.7)	55 (56.7)	29 (63.0)	0.47
BMI (kg/m^2)	21.1 ± 3.1	20.8 ± 3.1	21.8 ± 3.0	0.07
Hb (g/dL)	11.9 ± 2.2	12.3 ± 2.1	11.0 ± 2.2	<0.01**
BNP (pg/mL)	410.0 (185.4–836.6)	318.0 (180.3-589.6)	658.5 (304.7-1254.2)	<0.01**
Cr (mg/dL)	1.65 ± 1.24	1.68 ± 1.24	1.58 ± 1.24	0.64
eGFR (mL/min/1.73m^2)	39.6 ± 19.9	36.7 ± 16.4	45.6 ± 24.9	0.03*
Alb (g/dL)	3.44 ± 0.58	3.65 ± 0.44	3.01 ± 0.58	<0.01**
Preoperative Clinical Background				
NYHA [n (%)]				<0.01**
I	5 (3.5)	5 (5.2)	0 (0.0)	
II	43 (30.1)	42 (43.3)	1 (2.2)	
III	35 (24.5)	28 (28.9)	7 (15.2)	
IV	60 (42.0)	22 (22.7)	38 (82.6)	
Type of MR [n (%)]				0.23
FMR	110 (76.9)	74 (76.3)	36 (78.3)	
DMR	19 (13.3)	11 (11.3)	8 (17.4)	
Mixed	14 (9.8)	12 (12.4)	2 (4.3)	
Severity of Preoperative MR [n (%)]				0.24
1+	0 (0.0)	0 (0.0)	0 (0.0)	
2+	3 (2.1)	2 (2.1)	1 (2.2)	
3+	31 (22.0)	25 (26.0)	6 (13.3)	
4+	107 (75.9)	69 (71.9)	38 (84.4)	
STS score for MVR (%)	11.0 ± 7.4	9.88 ± 6.18	14.27 ± 9.40	0.02*
Preoperative LVEF (%)	37.8 ± 15.8	39.0 ± 15.4	35.2 ± 16.4	0.18
Preoperative LAD (mm)	50.1 ± 8.3	50.7 ± 8.1	48.8 ± 8.7	0.22
Preoperative LVEDD (mm)	60.8 ± 11.3	61.1 ± 12.5	60.3 ± 8.2	0.66
Procedural background				
Urgency [Planned (%)]	91 (63.6)	51 (53.7)	40 (95.2)	<0.01**
Follow-up outcome				
Deaths during follow-up [n (%)]	42 (29.4)	31 (32.0)	11 (23.9)	0.92 †

Continuous variables were analyzed using the t-test or Mann–Whitney U test, as appropriate, and categorical variables were compared using the chi-square test or Fisher's exact test, as appropriate. †The p value for deaths during follow-up was calculated using the log-rank test. Data are presented as mean ± standard deviation, median (interquartile range), or n (%), as applicable. *p<0.05, **p<0.01. Alb, albumin; BMI, body mass index; BNP, brain natriuretic peptide; Cr, creatinine; DMR, degenerative mitral regurgitation; eGFR, estimated glomerular filtration rate; FMR, functional mitral regurgitation; LAD, left atrial diameter; LVEDD, left ventricular end-diastolic diameter; LVEF, left ventricular ejection fraction; MR, mitral regurgitation; MVR, mitral valve replacement; NYHA, New York Heart Association; STS, Society of Thoracic Surgeons; TEER, transcatheter edge-to-edge repair.

Percentages were calculated using the number of available observations. The number of available observations was 143 unless otherwise indicated: Hb, n=142; albumin, n=137; type of MR, n=142; severity of preoperative MR, n=141; STS score for MVR, n=128; and procedure urgency, n=137.

Table S2. Causes of Death During Follow-up.

Cause of death	n (%)
**Cardiovascular death**	**14 (45.2)**
Heart failure	11 (78.6)
Other cardiovascular death	3 (21.4)
**Non-cardiovascular death**	**8 (25.8)**
Pneumonia/infection	1 (12.5)
Malignancy	5 (62.5)
Other non-cardiovascular death	2 (25.0)
**Unknown/undetermined**	**9 (29.0)**

Data are presented as n (% of deaths). Causes of death were classified according to the principal cause documented in medical records, referral letters, or information obtained from family members. Deaths for which the principal cause could not be determined were classified as unknown/undetermined.

Table S3. Sensitivity analysis including patients who died within 7 days after TEER and had available preoperative gait speed data.

Factor	Univariable analysis		Multivariable analysis (model 1)		Multivariable analysis (model 2)		Multivariable analysis (model 3)	
	Hazard ratio	P value	Hazard ratio	P value	Hazard ratio	P value	Hazard ratio	P value
Baseline Characteristics								
Age	1.06 (1.00-1.12)	0.02*					1.03 (0.98-1.08)	0.33
Sex	1.96 (0.96-4.03)	0.07						
BMI	0.91 (0.81-1.03)	0.13						
BMI Category								
Low Weight (<18.5)	1.99 (0.93-4.27)	0.08						
Obesity (≧25)	0.99 (0.23-4.32)	0.99						
Hb	0.71 (0.57-0.89)	<0.01**					0.76 (0.58-0.99)	0.04
BNP	1.00 (1.00-1.00)	0.63						
Cr	1.21 (0.98-1.49)	0.07						
eGFR	0.97 (0.95-0.99)	0.02*					0.98 (0.95-1.01)	0.13
Alb	0.55 (0.27-1.13)	0.12						
Preoperative Physical and Cognitive Function								
Gait Speed (per 0.1m/s)	0.70 (0.59-0.82)	<0.01**	0.73 (0.62-0.86)	<0.01**	0.78 (0.65-0.93)	<0.01**	0.72 (0.57-0.90)	<0.01**
Grip Strength	0.92 (0.88-0.97)	<0.01**			0.96 (0.91-1.01)	0.12		
MMSE	0.93 (0.85-1.01)	0.09						
CFS	1.55 (1.21-2.00)	<0.01**					1.09 (0.51-2.33)	0.58
Preoperative Clinical Background								
NYHA	1.16 (0.77-1.74)	0.48						
Type of MR		0.60						
FMR	1.00 (reference)	-						
DMR	0.51 (0.12-2.16)	0.36						
Mixed	1.23 (0.36-4.18)	0.74						

STS score for MVR	1.10 (1.04-1.15)	<0.01**	1.07 (1.01-1.13)	0.02*	1.07 (1.01-1.13)	0.03*		
Severity of Preoperative MR	2.03 (0.89-4.64)	0.09						
Preoperative LVEF	1.01 (0.99-1.03)	0.50						
Preoperative LAD	1.04 (0.99-1.09)	0.09						
Preoperative LVEDD	1.01 (0.98-1.05)	0.39						
Operative Details and Postoperative Cardiac Function								
Operation Time	1.01 (1.00-1.02)	0.01*						
Urgency	1.28 (0.63-2.60)	0.49					1.09 (0.51-2.33)	0.82
Blood Loss	1.01 (1.00-1.02)	0.09						
Postoperative LVEF	1.01 (0.99-1.04)	0.25						
Postoperative LAD	1.04 (1.00-1.09)	0.07						
Postoperative LVEDD	1.00 (0.97-1.03)	0.83						
Postoperative Hospitalization Details								
Hospital Stay	1.04 (1.03-1.06)	<0.01**						
Outcome	6.81 (2.81-16.51)	<0.01**						
Days to Standing	1.11 (1.00-1.23)	0.06						
Days to Walking	1.07 (1.02-1.09)	<0.01**						
Days to 100-meter Walking	1.06 (1.00-1.12)	0.05						
Continuous Walking Distance	1.00 (0.99-1.00)	0.01*						

Hazard ratios are presented with 95% confidence intervals. This sensitivity analysis included the two patients who died within 7 days after TEER but had available preoperative gait speed data. Model 1 was adjusted for the STS score for MVR; Model 2, for the STS score for MVR and handgrip strength; and Model 3, for age, hemoglobin, eGFR, procedure urgency, and CFS. Gait speed was analyzed per 0.1 m/s increase. Likelihood-ratio tests showed that adding gait speed improved model fit in Model 1 ($\Delta\chi^2 = 10.04$, df = 1, p = 0.002), Model 2 ($\Delta\chi^2 = 5.41$, df = 1, p = 0.020), and Model 3 ($\Delta\chi^2 = 8.45$, df = 1, p = 0.004). Univariable Cox regression analyses were performed using available data for each variable. Missing values were not imputed. CFS, Clinical Frailty Scale; eGFR, estimated glomerular filtration rate; Hb, hemoglobin; HR, hazard ratio; MVR, mitral valve replacement; STS, Society of Thoracic Surgeons; TEER, transcatheter edge-to-edge repair.

Figure S1. Kaplan–Meier curve for all-cause mortality. TEER, transcatheter edge-to-edge repair.

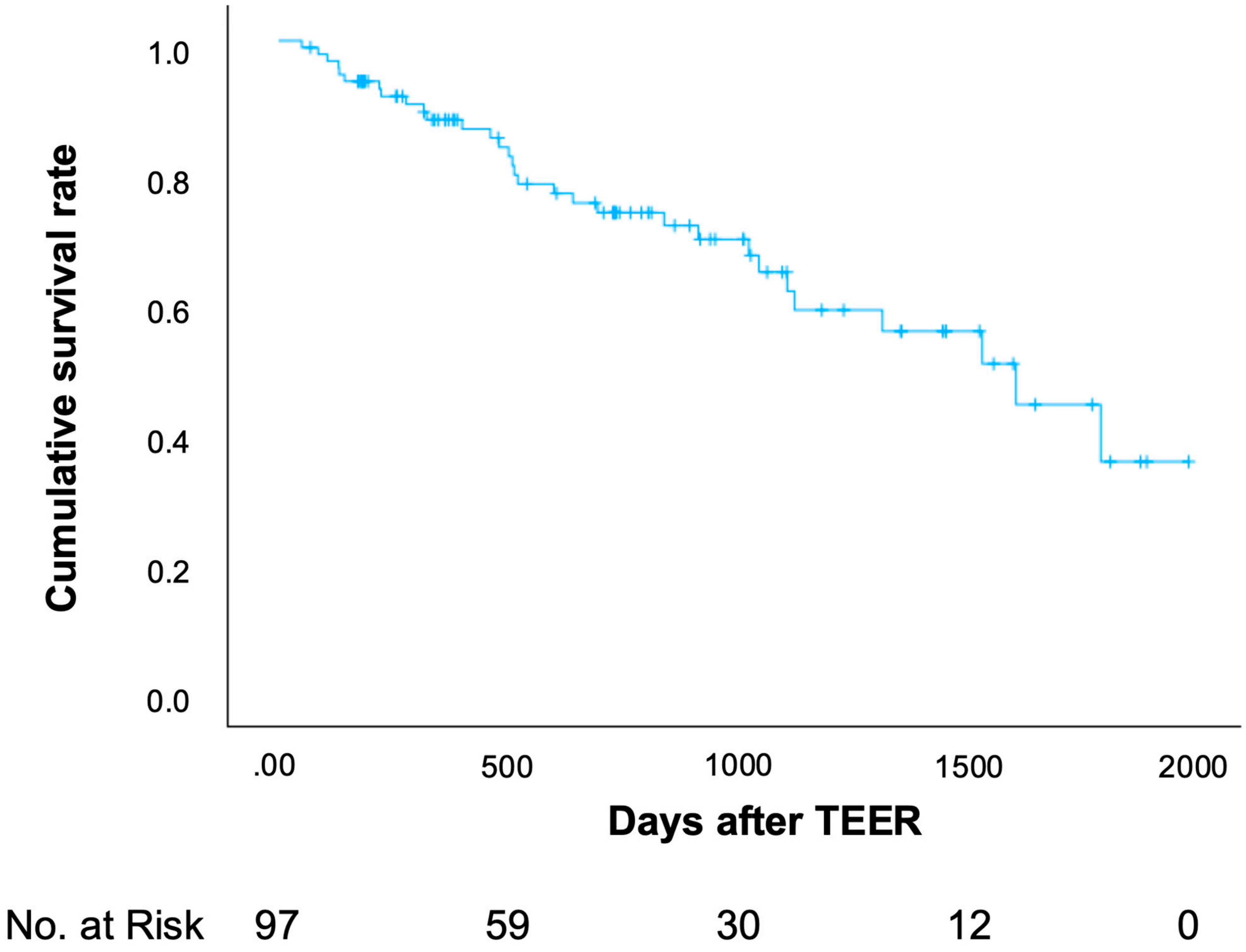

Figure S2. Kaplan–Meier curves for all-cause mortality according to availability of preoperative gait speed assessment among patients surviving beyond 7 days after TEER. Kaplan–Meier curves comparing all-cause mortality between patients included in the final analysis cohort and those in whom preoperative gait speed could not be assessed, after excluding patients who died within 7 days after TEER. TEER, transcatheter edge-to-edge repair.

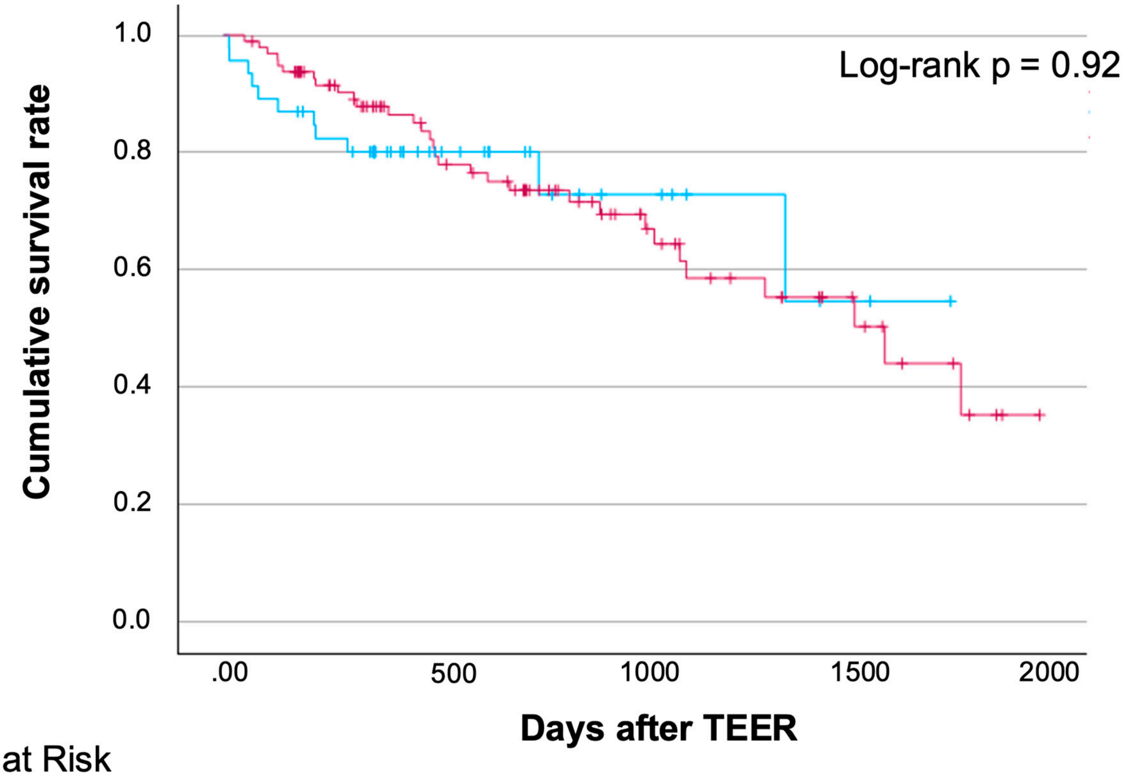

No. at Risk

Study patients	97	59	30	12	0
Gait speed-unavailable subgroup	46	20	7	2	0
